# *In vitro *evaluation of antibiotics' combinations for empirical therapy of suspected methicillin resistant *Staphylococcus aureus *severe respiratory infections

**DOI:** 10.1186/1471-2334-7-111

**Published:** 2007-09-21

**Authors:** Lorenzo Drago, Elena De Vecchi, Lucia Nicola, Maria Rita Gismondo

**Affiliations:** 1 Laboratory of Clinical Microbiology, Department of Preclinical Sciences LITA Vialba, University of Milan, Via GB Grassi 74, 20157 Milan, Italy; 2 Laboratory of Clinical Microbiology, Department of Clinical Sciences L. Sacco, University of Milan, Via GB Grassi 74, 20157 Milan, Italy

## Abstract

**Background:**

Methicillin resistant *Staphylococcus aureus *(MRSA) is an increasingly common cause of nosocomial infections, causing severe morbidity and mortality worldwide, and accounting in some hospitals for more than 50% of all *S. aureus *diseases. Treatment of infections caused by resistant bacterial pathogens mainly relies on two therapeutic modalities: development of new antimicrobials and use of combinations of available antibiotics.

Combinations of antibiotics used in the empiric treatment of infections with suspected methicillin resistant *Staphylococcus aureus *etiology were investigated.

**Methods:**

Double (vancomycin or teicoplanin with either levofloxacin or cefotaxime) and triple (vancomycin or teicoplanin + levofloxacin + one among amikacin, ceftazidime, cefepime, imipenem, piperacillin/tazobactam) combinations were evaluated by means of checkerboard assay and time kill curves. Mutational rates of single and combined drugs at antimicrobial concentrations equal to the resistance breakpoints were also calculated.

**Results:**

Vancomycin or teicoplanin + levofloxacin showed synergy in 16/50 and in 9/50 strains respectively, while vancomycin or teicoplanin + cefotaxime resulted synergic for 43/50 and 23/50 strains, respectively. Triple combinations, involving teicoplanin, levofloxacin and ceftazidime or piperacillin/tazobactam gave synergy in 20/25 strains. Teicoplanin + levofloxacin gave synergy in triple combinations more frequently than vancomycin + levofloxacin.

For single antibiotics, mutational frequencies ranged between 10^-5 ^and <10^-9 ^for levofloxacin, cefotaxime, amikacin and imipenem, and <10^-9 ^for vancomycin and teicoplanin. When tested in combinations, mutational frequencies fell below 10^-9 ^for all the combinations.

**Conclusion:**

*In vitro *evidence of synergy between glycopeptides, fluoroquinolones (levofloxacin) and β-lactams and of reduction of mutational frequencies by combinations are suggestive for a potential role in empirical therapy of severe pneumonia with suspected MRSA etiology.

## Background

Prevalence of bacterial pathogens resistant to the available antibiotics has been increasing over the past several decades. This feature represents a major challenge for developing innovative therapeutic modalities, particularly for treatment of Gram-positive infections. Among these, methicillin resistant *Staphylococcus aureus *(MRSA) is an increasing common cause of nosocomial infections, causing severe morbidity and mortality worldwide, and accounting in some hospitals for more than 50% of all *S. aureus *diseases [1-4]. Methicillin resistance was firstly identified in 1961 among nosocomial isolates of *S. aureus *and, till the last 10 years, was mainly restricted to the nosocomial setting [5]. However in the past decade new strains of MRSA in the community have been increasingly isolated reaching even 30% in some areas [6,7]. Of particular concern is that methicillin resistance, particularly in nosocomial strains, is often associated with resistance to other antibiotics such as macrolides and fluoroquinolones, so that glycopeptides and the oxazolidinones constitute the only available therapeutic options, once an MRSA infection has been diagnosed.

In the last years new antimicrobials specifically targeted against Gram-positive or MRSA strains have been launched, but strains with decreased susceptibility to these antibiotics have been increasingly isolated and, unfortunately, development of new antimicrobial agents in the next future seems to be declining [8-10]. On the other hand, antimicrobial combination therapy should be useful in improving efficacy, providing broad-spectrum coverage and preventing the emergence of resistant mutants [11]. Moreover, combination therapy is argued when empirical treatment of severe infections is needed, such as in severe pneumonia. As an etiological diagnosis with susceptibility results is almost never available to assist in the selection of the prompt therapy, the initial approach to treatment is largely empirical and is ordinarily guided by consensus guidelines. Moreover, early administration of appropriate empirical therapy for severe pneumonia has been demonstrated to significantly improve survival [12]. For this reason, antimicrobial therapy should cover both Gram-positive and Gram-negative bacteria, until obtainment of laboratory results. Guidelines from the American Thoracic Society suggest to add an anti-MRSA agent for treatment of pneumonia when MRSA is suspected, so that double or triple antimicrobial combinations are often used [13].

Despite their wide use, antimicrobial activity of antibiotics' combinations, particularly when more than two drugs are associated, is only rarely assessed in *in vitro *assays.

This study has been addressed to rate the interaction between glycopeptides (vancomycin and teicoplanin) and a β-lactam (cefotaxime) or a fluoroquinolone (levofloxacin) and among glycopeptides, a fluoroquinolone (levofloxacin) and either an aminoglycoside (amikacin) or a β-lactam (cefepime, ceftazidime, imipenem, piperacillin/tazobactam), which are suggested for empirical combinations in the hospital setting when a MRSA etiology is suspected [13]. Moreover, frequency of spontaneous mutations for single and combined antibiotics were also evaluated.

## Methods

### Microorganisms

Fifty MRSA clinical strains were isolated from low respiratory tract infections in patients hospitalized at L. Sacco Teaching Hospital of Milan, Italy. Isolates were identified using conventional automated methodologies (Vitek 2, BioMerieux, Marcy L'Etoile, France). Methicillin resistance was confirmed by oxacillin disk test in accordance with CLSI (formerly NCCLS) and Nitrocefin test [14,15]. Only one isolate for patient was considered in order to avoid duplicates. All isolates were stored in brain-heart infusion broth containing 10% (w/v) glycerol at -80°C until use.

### Antibiotics

Levofloxacin (LVX), cefotaxime (CTX), teicoplanin (TEC) (Sanofi-Aventis), amikacin (AMK), vancomycin (VA) (Sigma Aldrich), cefepime (FEP) (Bristol Myers Squibb), ceftazidime (CAZ) (Glaxo Smith Kline), imipenem (IPM) (Merck Sharp & Dohme) and piperacillin/tazobactam (TZP) (Wyeth Lederle) as powder of stated potency, were used to prepare stock solutions at concentrations of 5120 mg/L as suggested by CLSI [14,15].

### Determination of MIC

Determination of MIC was performed by means of microdilution broth method (microwell method) in accordance to CLSI [14,15]. *S. aureus *ATCC 29213 was used as quality control.

### Evaluation of synergy

Double combinations comprised a glycopeptide (VA or TEC) with either LVX or CTX. Triple combinations were composed by glycopeptide (VA or TEC), fluoroquinolone (LVX) plus either an aminoglycoside (AMK) or a β-lactam (FEP, CAZ, IPM, TZP).

#### Checkerboard

MICs of each antibiotic alone or in combination were determined by broth microdilution technique in accordance to CLSI standards by using cation adjusted Mueller-Hinton broth as modified for a broth microdilution checkerboard procedure [14-16]. For the double combinations a two-dimensional checkerboard with twofold dilutions of each drug was used for the study. The triple combinations were tested by a three-dimensional checkerboard technique in the following manner. A checkerboard with twofold dilutions of LVX and either VA or TEC was set up as described above for the double combinations. The third component of the combination was added at a single concentration per plate. Growth control wells containing medium were included in each plate. Each test was performed in duplicate. For the first clear well in each row of the microtiter plate containing all antimicrobial agents, the fractional inhibitory concentration (FIC) was calculated as follows: FIC of drug A (FIC_A_) = MIC of drug A in combination/MIC of drug A alone, FIC of drug B (FIC_B_) = MIC of drug B in combination/MIC of drug B alone, FIC of drug C (FIC_C_) = MIC of drug C in combination/MIC of drug C alone [17]. FIC Index (FICi), calculated as the sum of each FIC, was interpreted as follows: FICi ≤ 0.5 = synergy, FICi > 4.0 = antagonism and FICi > 0.5–4 = no interaction [18].

Double combinations were tested on 50 MRSA strains, triple combinations on 25 MRSA strains.

#### Time-kill curves

Time kill curve were performed on five randomly selected MRSA strains, among those showing the lowest MICs. Time-kill assays were performed in Mueller Hinton broth inoculated with each isolate to a final concentrations of 1–5 × 10^5 ^cfu/mL.

Antibiotics were tested alone and in double combinations at concentrations equal to 1 × MIC, 1/2 × MIC, 1/4 × MIC or in triple combinations at concentrations of 1 × MIC, 1/2 × MIC, 1/4 × MIC and 1/8 × MIC. After 0, 2, 6, 12 and 24 hours of incubation, aliquots of bacterial culture were serially diluted and plated on to Mueller Hinton agar for colony counts.

Synergy was defined as a 2 log_10 _decrease in colony counts, when antibacterial activity of combinations was compared with that of the most active single agent. Indifference was defined as <2 log_10 _increase in colony count at 24 h by the combination compared by the most active single agent. Antagonism was defined as a ≥2 log_10 _increase in colony count at 24 h by the combination compared with that by the most active single agent alone [17].

### Mutational frequency

The frequency of spontaneous single-step mutations was determined on 10 strains of MRSA by spreading 0.1 mL from a bacterial suspension of about 10^10 ^CFU/mL on antibiotic free agar plates (after proper dilution) and on antibiotic containing agar plates (undiluted inoculum) [19]. CLSI resistance breakpoints were used to define antibiotic concentrations in each plate [15]. Colonies grown after 48 h of incubation at 37°C were counted. Frequency of mutation was calculated as the number of colonies grown on antibiotic containing plates per inoculum. Only antibiotics active against the chosen strains were tested alone.

## Results

### MIC

Microbial susceptibilities to the tested antibiotics are shown in Table 1. All the strains were resistant to cefotaxime, and most of them were resistant also to levofloxacin, while they were all susceptible to vancomycin and teicoplanin. Three strains were found resistant to amikacin, while no appreciable activity against MRSA was observed for ceftazidime, cefepime, imipenem and piperacillin/tazobactam.

**Table 1:** Antimicrobial activity of antibiotics tested in the study on clinical MRSA strains

	**Number of strains having MIC (μg/ml) equal to:**														
	
	**2048**	**1024**	**512**	**256**	**128**	**64**	**32**	**16**	**8**	**4**	**2**	**1**	**0.5**	**0.25**	**0.125**
**LVX**							3 (1)*	5 (2)	35 (17)	3 (3)		4 (2)			
**CTX**	34	3	5	2	2	2	2								
**VA**										3 (1)	9 (3)	20 (13)	18 (8)		
**TEC**										2 (1)	3 (1)	23 (12)	12 (5)	6 (3)	4 (3)
**AMK**				1	2		2	3	10	6		1			
**CAZ**		7	13	2	3										
**FEP**		15	4		2	4									
**IPM**			5	13	3	2					2				
**TZP**			3	14	2	2	2	1	1						

*: Data in parenthesis refer to strains used in triple combinations; LVX: levofloxacin; CTX: cefotaxime; VA: vancomycin; TEC: teicoplanin; AMK: amikacin; CAZ: ceftazidime; FEP: cefepime; IPM imipenem; TZP: piperacillin/tazobactam.

### Evaluation of synergy

#### Checkerboard

As shown in Table 2, all combinations showed synergy or no interaction, while no antagonism was found in the checkerboard assays. In particular, among double combinations CTX + TEC gave the highest rate of synergy (43/50), followed by CTX + VA (23/50), LVX+ VA (16/50) and LVX + TEC (9/50).

**Table 2:** Interaction as determined by means of checkerboard assay

**Combination**	**Nr of MRSA strains (%) for which combinations gave**		
	
	**Synergy**	**No interaction**	**Antagonism**
**LVX + VA (n = 50)**	16 (32)	34 (68)	0
**LVX + TEC (n = 50)**	9 (18)	41 (82)	0
**CTX + VA (n = 50)**	23 (46)	27 (54)	0
**CTX + TEC (n = 50)**	43 (86)	7 (14)	0
**LVX + VA + AMK (n = 25)**	5 (20)	20 (80)	0
**LVX + TEC + AMK (n = 25)**	10 (40)	15 (60)	0
**LVX + VA + CAZ (n = 25)**	15 (60)	10 (40)	0
**LVX + TEC + CAZ (n = 25)**	20 (80)	5 (20)	0
**LVX + VA + FEP (n = 25)**	10 (40)	15 (60)	0
**LVX + TEC + FEP (n = 25)**	10 (40)	15 (60)	0
**LVX + VA + IPM (n = 25)**	5 (20)	20 (80)	0
**LVX + TEC + IPM (n = 25)**	10 (40)	15 (60)	0
**LVX + VA + TZP (n = 25)**	5 (20)	20 (80)	0
**LVX + TEC + TZP (n = 25)**	20 (80)	5 (20)	0

LVX: levofloxacin; VA: vancomycin; TEC: teicoplanin; CTX: cefotaxime; AMK: amikacin; CAZ: ceftazidime; FEP: cefepime; IPM: imipenem; TZP: piperacillin/tazobactam.

Triple combinations of levofloxacin resulting synergic on the highest number of strains involved TEC with either CAZ or TZP (20/25 strains), while combinations with TEC and either AMK or FEP or IPM yielded synergy in 10/25 strains. Among the combinations of LVX with VA, the highest rate of synergy was observed in those with CAZ (15/25), followed by combinations with FEP (10/25) and with AMK or IMP or TZP which resulted synergic in a small portion of the tested MRSA strains (5/25).

#### Time-kill curves

In double combinations, synergy occurred earlier with combinations involving TEC/VA and CTX. As shown in table 3, TEC/VA + CTX showed synergy in all the tested strains after 12 h, while the same result was obtained with LVX combinations after 24 h.

**Table 3:** Evaluation of synergy: time killing curves on 5 MRSA strains

**Combination**	**Nr of strains showing synergy/indifference at:**			
	
	**2 h**	**6 h**	**12 h**	**24 h**
**LVX + VA**	0/5	0/5	2/3	5
**LVX + TEC**	0/5	0/5	3/2	5/0
**CTX + VA**	0/5	1/4	5/0	5/0
**CTX + TEC**	0/5	0/5	5/0	5/0
**LVX + VA + AMK**	0/5	0/5	0/5	3/2
**LVX + TEC + AMK**	0/5	0/5	0/5	4/1
**LVX + VA + CAZ**	0/5	0/5	0/5	2/3
**LVX + TEC + CAZ**	0/5	1/4	2/3	3/2
**LVX + VA + FEP**	0/5	0/5	0/5	3/2
**LVX + TEC + FEP**	0/5	0/5	1/4	3/2
**LVX + VA + IPM**	0/5	0/5	1/4	3/2
**LVX + TEC + IPM**	0/5	1/4	2/3	3/2
**LVX + VA + TZP**	0/5	0/5	2/3	2/3
**LVX + TEC + TZP**	0/5	0/5	3/2	3/2

LVX: levofloxacin; VA: vancomycin; TEC: teicoplanin; CTX: cefotaxime; AMK: amikacin; CAZ: ceftazidime; FEP: cefepime; IPM: imipenem; TZP: piperacillin/tazobactam.

Triple combinations of TEC with LVX generally showed an overall high degree of synergy if compared with combinations with VA. Combinations of TEC with LVX and β-lactams gave synergy against at least 1 out of the 5 strains tested after 12 h, while at the same time LVX + VA showed synergy only with IPM and TZP. After 24 h, combinations with TEC showed synergy for 3/5 strains when the third antibiotic was a β-lactam and for 4/5 strains when combined with AMK. At the same time, combinations with VA were synergic for 2/5 strains with CAZ and TZP and for 3/5 strains when LVX and VA were combined with AMK, FEP and IPM.

No antagonistic effects were observed with all the tested combinations, while indifference was found for the all the other strains.

### Mutational frequency

For single antibiotics, mutational frequencies at breakpoint antibiotic concentrations ranged between 10^-5 ^and <10^-9 ^for LVX, CTX, AMK and IMI, and were <10^-9 ^for VA and TP, while they were not determined for CAZ, CPM and PTZ since none of the tested strains was susceptible to these drugs (Tables 4 and 5). When tested in double and triple combinations, mutational frequencies fell below 10^-9 ^for all the tested antibiotics.

**Table 4:** Mutational frequencies of levofloxacin, vancomycin, teicoplanin and cefotaxime alone and in double combination

**Strain**	**Single antibiotics**				Double combinations*
	**LVX**	**VA**	**TEC**	**CTX**	

**7095**	<10^-9^	<10^-9^	<10^-9^	n.d.	<10^-9^
**7096**	<10^-9^	<10^-9^	<10^-9^	n.d.	<10^-9^
**8957**	1.65 × 10^-5^	<10^-9^	<10^-9^	n.d.	<10^-9^
**7176**	1.81 × 10^-6^	<10^-9^	<10^-9^	n.d.	<10^-9^
**7171**	<10^-9^	<10^-9^	<10^-9^	n.d.	<10^-9^
**2106**	<10^-9^	<10^-9^	<10^-9^	n.d.	<10^-9^
**6556**	2.86 × 10^-7^	<10^-9^	<10^-9^	5.47 × 10^-5^	<10^-9^
**7169**	n.d.	<10^-9^	<10^-9^	1.22 × 10^-5^	<10^-9^
**7955**	n.d.	<10^-9^	<10^-9^	n.d.	<10^-9^
**8363**	n.d.	<10^-9^	<10^-9^	n.d.	<10^-9^

LVX: levofloxacin; VA: vancomycin; TEC: teicoplanin; CTX: cefotaxime.

n.d.: not determined (strain resistant to the antibiotic)

*Double combinations: LVX + VA; LVX + TP; CTX + VA; CTX + TP

**Table 5:** Mutational frequencies of levofloxacin, vancomycin, teicoplanin, amikacin, ceftazidime, cefepime, imipenem and piperacillin/tazobactam alone and in triple combinations

**Strain**	**LVX**	**VA**	**TEC**	**AMK**	**CAZ**	**FEP**	**IPM**	**TZP**	Triple combinations*
**7095**	<10^-9^	<10^-9^	<10^-9^	4.52 × 10^-7^	n.d.	n.d.	n.d.	<10^-9^	<10^-9^
**7096**	<10^-9^	<10^-9^	<10^-9^	1.6 × 10^-7^	n.d.	n.d.	1.03 × 10^-6^	n.d.	<10^-9^
**8957**	4.9 × 10^-5^	<10^-9^	<10^-9^	<10^-9^	n.d.	n.d.	n.d.	n.d.	<10^-9^
**7176**	2.4 × 10^-6^	<10^-9^	<10^-9^	3.65 × 10^-8^	n.d.	n.d.	8.52 × 10^-5^	n.d.	<10^-9^
**7171**	<10^-9^	<10^-9^	<10^-9^	7.57 × 10^-7^	n.d.	n.d.	n.d.	n.d.	<10^-9^
**2106**	n.d.	<10^-9^	<10^-9^	<10^-9^	n.d.	n.d.	2.3 × 10^-5^	n.d.	<10^-9^
**7117**	n.d.	<10^-9^	<10^-9^	7.24 × 10^-7^	n.d.	n.d.	n.d.	n.d.	<10^-9^
**7169**	n.d.	<10^-9^	<10^-9^	<10^-9^	n.d.	n.d.	n.d.	n.d.	<10^-9^
**6491**	n.d.	<10^-9^	<10^-9^	6 × 10^-8^	n.d.	n.d.	n.d.	n.d.	<10^-9^

LVX: levofloxacin; VA: vancomycin; TEC: teicoplanin; AMK: amikacin; CAZ: ceftazidime; FEP: cefepime; IPM: imipenem; TZP: piperacillin/tazobactam.

n.d.: not determined (strain resistant to the antibiotic)

*Triple combinations: LVX+VA+AMK; LVX+VA+CAZ; LVX+VA+FEP; LVX+VA+IPM; LVX+VA+TZP; LVX+VA+AMK; LVX+TEC+CAZ; LVX+TEC+FEP; LVX+TEC+IPM; LVX+TEC+TZP.

## Discussion

Antimicrobial combination therapy may be used to extend spectrum coverage, prevent the emergence of resistant mutants and gain synergy between antimicrobials [11]. Combination therapy is often recommended for empirical treatment of bacterial infections in intensive care units, where monotherapy is not likely to cover all potential pathogens, and the emergence of resistance is a potential threat [20]. In the last years, development of new antimicrobials targeted to treatment of MRSA infections, such as linezolid, has led to assess antibacterial activity of such drugs in combination with several antibacterials, particularly β-lactams [21-24]. Also combination of vancomycin with aminoglycoside or β-lactams has been widely investigated, generally reporting synergy with β-lactams while no interactions of even antagonism were observed with aminoglycosides [25-28]. Activity of combinations containing teicoplanin against *S. aureus *has been less investigated [27,28]. The present study has compared antimicrobial combinations containing teicoplanin or vancomycin for their antibacterial activity and ability to select for mutants able to grow in their presence in nosocomial MRSA. To our knowledge this is the first study evaluating the efficacy of triple combinations of a glycopeptide, a β-lactam and a fluoroquinolone against MRSA strains. Triple combinations with vancomycin have been recently indicated by American Thoracic Society and Infectious Diseases Society of America for initial empiric therapy for hospital acquired pneumonia, ventilator associated pneumonia and healthcare associated pneumonia in patients with potential MRSA etiology [13]. These guidelines suggest the use of vancomycin or, as alternative, of linezolid, combined with one among antipseudomonal cephalosporins or carbapenems or β-lactam/β-lactamase inhibitor (i.e. piperacillin-tazobactam) and an antipseudomonal fluoroquinolone or an aminoglycoside. Teicoplanin is not included, since it has not been approved in the United States, however, in all countries where it has been approved, teicoplanin represents an alternative to vancomycin [13]. In this study, data from the checkerboard assay indicates that teicoplanin with cefotaxime showed the highest rate of synergy among the tested combinations, followed by those with levofloxacin and ceftazidime or piperacillin/tazobactam. Ability of β-lactams in triple combinations to yield synergy was quite different for the various molecules, ranging from 20 to 80%.

Killing curves confirmed superior activity of teicoplanin plus cefotaxime, while smoothed differences among β-lactams. Since in *in vitro *experiments glycopeptides are slowly bactericidal, it may be hypothesized that their antistaphylococcal bactericidal activity could be increased by use of combinations [29]. It is also evident that this ability was not a class effect since marked differences were found among β-lactams tested and between the two glycopeptides. Therefore it seems rather difficult to compare our data with those of other studies in which different β-lactams have been used.

Methicillin resistance is known to favor development of multi-drug resistance, including macrolides and fluoroquinolones. In fact, for the strains evaluated in this study, determination of frequency of mutation for each antibiotic in single was not always possible, since they were already resistant to the molecules tested and therefore able to grow at antibiotic concentrations equal to the resistant breakpoint. Data obtained indicate that glycopeptides in double or triple combinations were effective in preventing the emergence of mutants able to grow in presence of the antibiotics. No mutants were isolated *in vitro *after incubation with glycopeptides alone, thus correlating the low frequency of development of glycopeptides resistance observed *in vivo*. Different results have been reported for teicoplanin and vancomycin after serial exposure to these antibiotics by other authors, but the differences between the methods employed are likely the cause of this discrepancy [30-33]. However, further studies on development of resistance possibly induced by the tested antibiotics in single and in combinations after serial passages are now in progress.

## Conclusion

In summary, double and triple combinations of glycopeptides with levofloxacin and/or β-lactams were studied as far as synergy and frequency of mutations were concerned in a congruent number of MRSA strains. High rates of synergy were obtained for some combinations including teicoplanin, while combinations with both glycopeptides reduced frequency of mutations to not detectable levels. Although *in vitro *studies need to be confirmed by *in vivo *findings, the data obtained support the use of antimicrobial combinations including glycopeptides and β-lactams in initial empirical therapy of severe pneumonia, potentially sustained by MRSA and non fermenting gram-negative bacteria, limiting the development of mutants able to grow in presence of antibiotics.

## Competing interests

LD received research funding from sanofi aventis.

## Authors' contributions

LD participated in design and coordination of the study, interpretation of the data, and co-drafted the manuscript. EDV participated in study design, co-performed killing curves and frequency of mutation assays, analysis and interpretation of the data and co-drafted the manuscript. LN performed checkerboard assays, time kill curves and frequency of mutation assays and participated in analysis and interpretation of the data. MRG participated in revising the manuscript.

All authors read and approved the final manuscript.

## Pre-publication history

The pre-publication history for this paper can be accessed here:


